# Cytogenetic/mutation profile of chronic lymphocytic leukemia/malignant melanoma collision tumors of the skin

**DOI:** 10.1186/s13039-017-0353-1

**Published:** 2018-01-16

**Authors:** Roberta La Starza, Tiziana Pierini, Lorenza Pastorino, Elisa Albi, Caterina Matteucci, Barbara Crescenzi, Paolo Sportoletti, Piero Covarelli, Franca Falzetti, Giovanni Roti, Stefano Ascani, Cristina Mecucci

**Affiliations:** 1 Molecular Medicine Laboratory, Hematology and Bone Marrow Transplantation Unit, University of Perugia, Hospital S. Maria della Misericordia, Piazzale Menghini n.9, 06132 Perugia, Italy; 2 Department of Internal Medicine and Medical Specialties (DiMI), University of Genova and IRCCS AOU San Martino-IST, Viale Benedetto XV n.6, 16132 Genova, Italy; 3 Department of Surgery, University of Perugia, Piazzale Menghini n.1, 06132 Perugia, Italy; 4 C.S. Ematology and Center of bone marrow transplants, University and Hospital of Parma, Via Gramsci n.14, Parma, 43126 Italy; 5 Institute of Pathology, University of Perugia and Hospital S. Maria di Terni, Viale Tristano di Joannuccio n.1, 05100 Perugia, Italy

**Keywords:** Collision tumor, CLL, Melanoma, Molecular cytogenetics, Mutational analysis

## Abstract

**Background:**

Collision tumors are rare entities that consist of two histologically distinct tumor types arising in the same anatomic site. An association between chronic lymphocytic leukemia (CLL) and malignant melanoma (MM) has been already described. Up to now, they have been documented only at positive regional lymph nodes while we focused on collision tumor in a skin lesion.

**Case presentation:**

We characterized the genomic profile of a skin CLL/MM collision tumor in a patient with a 9-years story of CLL. Typical high-grade genomic biomarkers featured the CLL: the immunoglobulin heavy variable genes were unmutated; a clonal del(11q), involving *ATM* and *BIRC3*, was present in the peripheral blood (PB) and skin lesion, while a subclonal large del(13q)/D13S319*-RB1* was detected only in the PB. Interestingly, the del(13q) clone, increased from 10% to 46% from diagnosis to relapse. *NOTCH1*, *SF3B1*, and *TP53* were wild type. The MM lesion carried a *BRAF*^V600E^ and a *TERT* promoter mutation.

As the family story was consistent with a genetic predisposition to cancer, we performed mutational analysis of genes involved in familial melanoma and CLL, and of *BRCA1* and *BRCA2*. No germinal mutation known to predispose to CLL, MM, or breast cancer was found. Interestingly, conventional cytogenetic detected a constitutional t(12;17)(p13;p13).

**Conclusions:**

Our data are consistent with distinct genetic landscape of the two tumors which were characterized by specific disease-related abnormalities. CLL cells carried poor prognostic imbalances, i.e. large deletions of the long arm of chromosomes 11 and 13, while in MM cells two functionally linked mutations, i.e. *BRAF*^V600E^ and a *TERT* promoter occurred. Although, known germline variations predisposing to MM and/or CLL were ruled out, genetic counseling suggested the proband family was at high risk for MM.

## Background

CLL, the most frequent hematological disease in adults, is characterized by a marked variable outcome, from an indolent clinical course to more aggressive forms with acquisition of chemo-resistance, after a benign onset. CLL evolution is largely dependent upon molecular and cytogenetic features which are well recognized prognostic markers [1, 2]. Of note, 30–35% of long-term CLL survivors are at high risk of developing secondary neoplasms mostly epithelial. This susceptibility do not appear to depend on anti-neoplastic treatment as the same incidence of leukemia was observed in treated and untreated patients [3]. Namely, CLL patients have a four-fold increase in the risk of developing MM compared with the general population.

MM and CLL collision tumors were reported, as occasional findings, in metastatic lymph nodes of patients with cutaneous MM [4, 5]. A collision tumor is defined as the occurrence of two neoplastic cell populations in close proximity to each other, though maintaining separate boundaries. Combination of solid and hematological neoplasms, such as colon or breast carcinoma or MM, in conjunction with non-Hodgkin lymphoma or CLL, are the most frequent association [6, 7].

We report the molecular-cytogenetic characterization of a unique case of CLL and MM collision tumors. Our molecular findings are consistent with an independent origin of the two tumors and suggest that both a familial predisposition and the CLL-associated immune dysregulation might have played a role in their onset.

## Case presentation

**Fig. 1:**
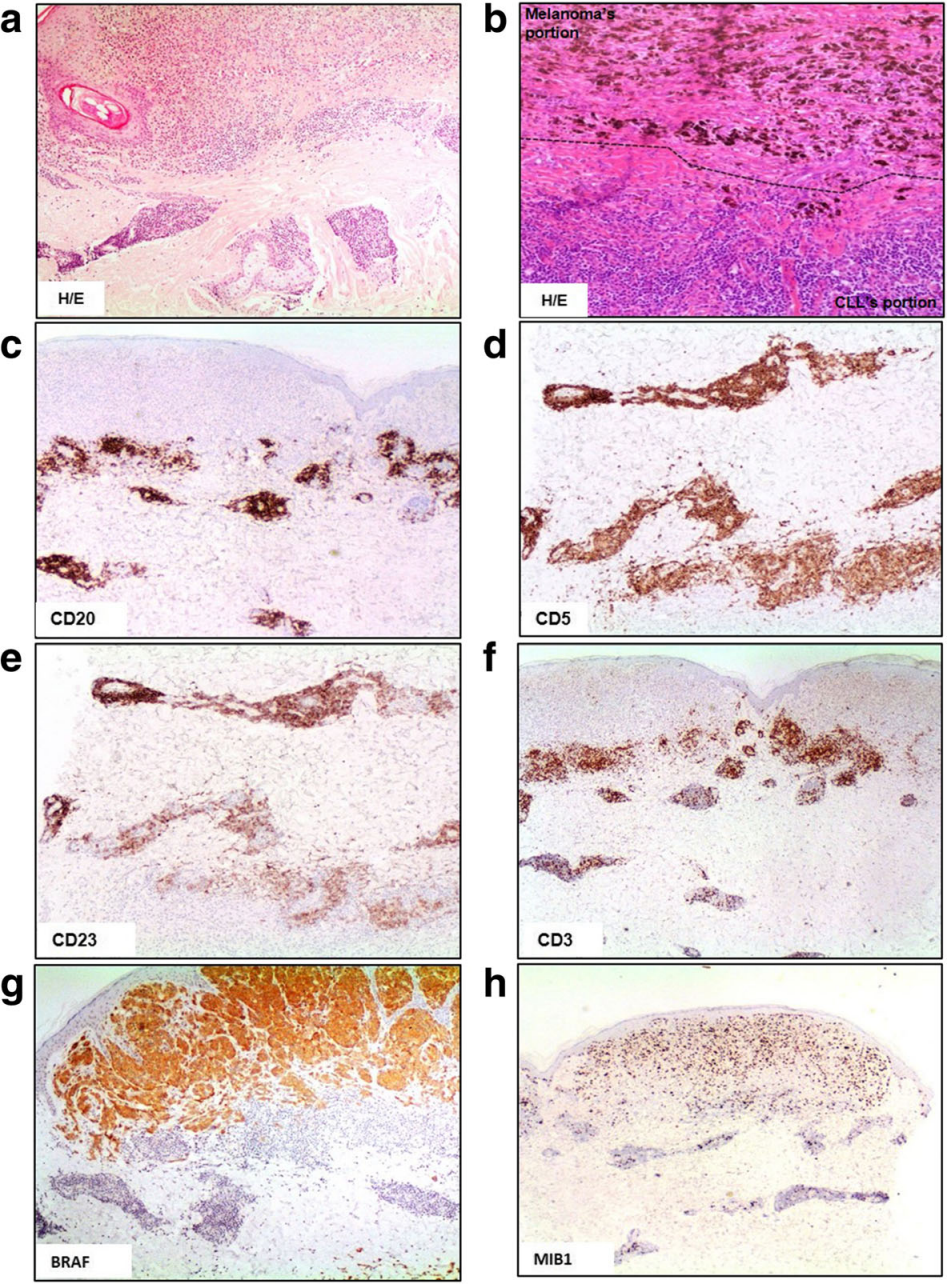
Immunohistochemical characterization of skin lesion. **a** Haematoxylin/eosin staining, ×4. **b** Haematoxylin/eosin staining, ×10, the dotted black line defines the border area between CLL and MM tumors. **c**, **d** and **e** Immunohistochemical positivity for CD20, CD5 and CD23 antigens. **f** Immunohistochemical negativity for CD3. **g** Presence of mutant BRAF only in MM lesion. **h** MIB1 expression to evaluate the neoplastic proliferation

At diagnosis of CLL (Rai stage I; Binet stage B), the patient, a 58 year-old male, had 20.150/mmc white blood cells with 75% lymphocytes. The bone marrow (BM) biopsy showed a diffuse pattern of infiltration by small CD20, CD5, CD23, CD38, and ZAP70 positive lymphoid elements with unmutated immunoglobulin heavy chain. The patient was treated with 6 cycles of fludarabine, cyclophosphamide, and rituximab, achieving hematological remission. He relapsed after 79 months from diagnosis with marked lymphocytosis (211.100/mmc) and widespread lymphadenopathy; PET scan assessed lymph nodes hyperactivity (SUV max 6.1). At this time, the patient presented a 17 × 13 mm skin lesion plus twenty-three ~5 mm lesions on the right side of the trunk. Histology and immunohistochemical features of the main lesion and in-transit metastasis were consistent with a diagnosis of collision CLL and MM tumors. The MM lesion infiltrated the epidermal layer and the reticulis dermis (Breslow thickness of 3,4 mm; mitosis 3/mm2) and showed pigmentation, regression, and ulceration. The MM was classified as stage T3N2M0. The underlying dermis, and partially the hypodermis, were infiltrated by small CD20, CD5, CD23 positive, CD3 and cyclin D1 negative lymphoid cells (Fig. 1).

MM treatment consisted of wide local excision of the primary lesion, surgical dissection of 71 axillary lymph nodes (all positive for CLL involvement), and electrochemotherapy with intravenous injection of bleomycin for three times, which elicit a short lasting complete remission (9 months) of skin lesions. CLL treatment consisted of 6 cycles of bendamustine and rituximab which induced the hematological remission of CLL.

At MM relapse, a combined treatment with BRAF (dabrafenib) and MEK (trametinib) inhibitors was started. He is still under treatment and in remission of MM skin lesions.

## Methods

### Genetic counseling

**Fig. 2:**
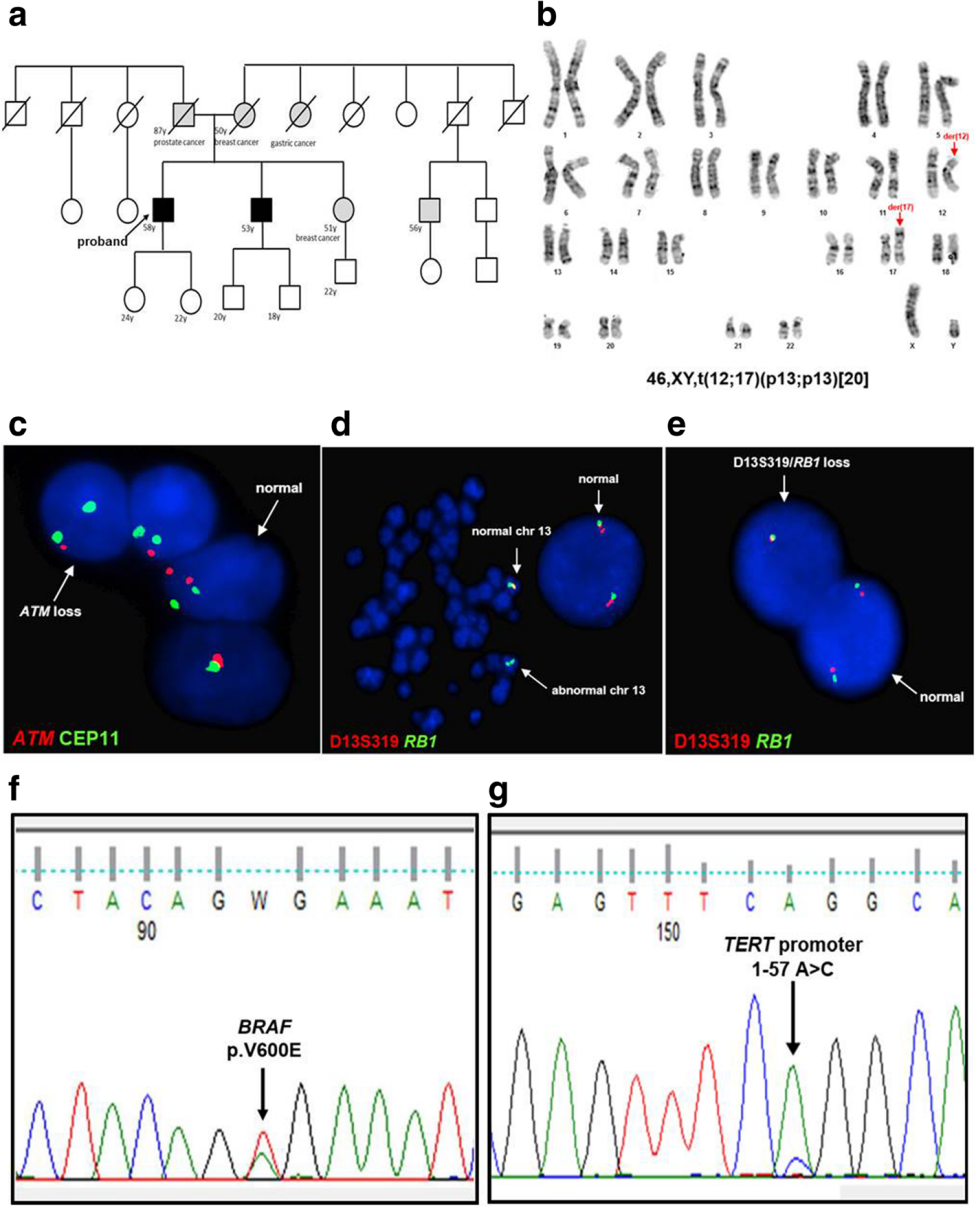
Clinical and molecular data of CLL/MM collision tumor. **a** Pedigree of patient’s family. The proband is indicated by a black arrow. **b** Constitutional patient karyotype showed the t(12;17)(p13;p13) in all metaphases analyzed [20]. The 12 and 17 derivative chromosomes are indicated by red arrows. **c** Interphase FISH shows *ATM* monoallelic deletion (red signal referred to target gene and green signal referred to centromere 11). The abnormal and normal nucleus are indicated by white arrows. **d** Metaphase FISH of the 13q14 region (D13S319) (red signal) and the *RB1* gene (green signal). The arrows indicate the abnormal derivative chromosome 13 with loss of D13S319 region, and the normal chromosome 13. **e** Interphase FISH of the 13q14 region (D13S319) (red signal) and the *RB1* gene (green signal). The white arrows show a nucleus with loss of both D13S319 region and *RB1* gene, and a nucleus without deletion. **f** Nucleotidic sequence of *BRAF* (exon 15) with hot-spot mutation (p.V600E) on melanoma lesion (black arrow). **g** Nucleotidic sequence of *TERT* promoter mutation (1–57 A > C) on melanoma lesion (black arrow)

The family story satisfied the criteria of familial melanoma as two first-degree relatives, the proband and his brother developed MM [8]. In particular, the brother was affected by 4 cutaneous MM. Additionally, the proband’s mother and sister had both suffered of breast cancer, at the age of 49 and 51, respectively (Fig. 2a). Genetic testing was performed by Ion Personal Genome Machine® (PGM™) Sequencer (Thermo Fisher Scientific Inc., Monza, Italy) using a custom familial melanoma panel including *BAP1*, *CDKN2A*, *CDK4*, *TERT* promoter (p*TERT*), *MITF* exon 10, *ATM*, *PALB2,* and *POT1*; *TERF2IP* and *ACD*, also known to be involved in familial CLL [9]. Sanger’s sequencing investigated *BRCA1* and *BRCA2* and validated next generation sequencing findings (3500 Genetic Analyzer-Life Technologies, Monza, Italy). Constitutional karyotype of the proband and his brother and sister, was done on phytohaemagglutinin (PHA) stimulated peripheral blood (PB) T-lymphocytes.

### Molecular-cytogenetic studies

All studies were carried out in accordance with the Declaration of Helsinki. Written informed consent was obtained from the patient for publication of clinical history and any additional related information. The study was approved by the Bioethics Committee of the University of Perugia (prot. Number 2014–019).

A specific CLL panel probes (LSI ATM SO/CEP11 SG*,* LSI TP53 SO/CEP17 SG, LSI D13S319/LSI 13q34/CEP12 Multi-color probe set, LSI 13 RB1 SO, LSI D13S319 SO and LSI IgH dual color break-apart; Vysis Abbott, Milan, Italy), was applied on PB samples taken at diagnosis and relapse, and on formalin-fixed paraffin embedded skin lesion after marking the MM and CLL areas according to haematoxylin/eosin staining. Home-made probes were also used to investigate the 11q region, by *BIRC3*/11q22.2 (RP11-605B8) and *DDX10*/11q22.3 (RP11-244G23), and the *RB1*/13q14.2 (RP11-305D15, RP11-174I10) (UCSC Genome Browser, Human/Feb.2009 GRCh37/hg19). Analysis was carried out on 200 nuclei/100 cells per experiment with fluorescence microscopy using an Olympus BX61 (Olympus, Milan, Italy) equipped with a JAI camera (Copenhagen, Denmark) and CytoVision 4.5.4 software (Genetix, New Milton, Hampshire, UK). CLL and MM areas were analyzed separately.

Hot-spot mutations of *BRAF,* p*TERT*, *SF3B1, NOTCH1*, and the whole coding region of *TP53* were studied by Denaturing High Performance Liquid Chromatography (DHPLC) and/or Sanger’s sequencing of genomic DNA samples extracted from skin biopsy after macro-dissection to separate MM from CLL.

## Results

FISH (fluorescent in situ hybridization) analysis of the CLL skin lesion detected a monoallelic del(11)(q23) with loss of a large 11q region, involving *ATM* and *BIRC3* (Fig. 2c). Besides del(11)(q23), PB samples also carried a del(13q). Double-color FISH assay combining probes for common deleted region D13S319 and *RB1* demonstrated that two distinct clones were present: 10% of cells had a small del(13q)/D13S319; 3% of cells had a large del(13q) involving both *RB1* and D13S319 (Fig. 2d, e). The two clones increased at relapse to 46% and 13%, respectively. The CLL-associated genomic rearrangements were not detected in the MM skin lesion. No mutations of *NOTCH1*, *SF3B1*, and *TP53* were found. The MM cells carried a *BRAF*^V600E^ and a p*TERT* c.1–57 A > C mutation.

The familial melanoma panel did not detect known predisposing gene variations. *BRCA1* and *BRCA2* were wild type.

## Discussion

This study reports, for the first time, a collision CLL/MM tumor of the skin, and the molecular-cytogenetic background underlying the two tumors. Collision tumors occurr very rarely, and the association of CLL and MM have been sporadically found, at metastatic lymph nodes, of patients with MM [4, 5].

Our molecular and cytogenetic studies showed that the CLL and the MM had distinct genetic landscapes. The CLL skin lesion and PB samples were wild type for recurrent CLL associated mutations and shared high risk biomarkers. Namely, unmutated *IgH* variable regions and monoallelic del(11)(q23). *ATM* is known to be involved in DNA repair processes that are altered when deletion and/or *ATM* loss-of-function mutations occur; *BIRC3*, a negative regulator of alternative NFkB signaling pathway, is implicated in the modulation of different cellular processes, such as apoptosis, cell proliferation, invasion and metastasis, inflammatory and mitogenic kinase signaling. Several lines of evidence indicate that *ATM* biallelic deletions and/or loss-of-function mutations are poor prognostic markers correlating with a significantly reduced overall survival, while *BIRC3* haplo-insufficiency does not appear to have a prognostic value [10].

Besides del(11)(q22-q23), PB samples also carried a del(13q), which is the most common cytogenetic aberration in CLL, occurring in ~40% of cases and, when isolated, and it’s regarded as good prognostic marker. However, the amount of leukemic cells bearing the del(13q) as well as the extension of the deleted region, appeared to influence patient’s outcome [11]. Moreover, large del(13q) which include the *RB1* oncosuppressor gene, called “type II” deletions, were associated with genome complexity [12]. In our case, FISH showed that both the D13S319 locus and *RB1* were lost as expected in cases with progressive disease and both clones increased at relapse (Fig. 2d, e).

Negative for CLL-associated genomic losses, the MM carried a *BRAF*^V600E^ and a p*TERT* c.1–57 A > C mutation (Fig. 2f, g). It is worth mentioning that p*TERT* mutations occur as germline, in familial MM, or acquired in sporadic MM [13]. The majority of p*TERT* mutations occurr at c.1–124 C > T and c.1–146 C > T positions, and both generate binding motifs for the Ets/TCF transcription factors. The p*TERT* c.1–57 A > C mutation, of our case, was previously reported in familial MM and in bladder tumors [13].

The *BRAF*^V600E^ is an early genomic lesion, occurring in melanocytic nevi, not sufficient to drive a full-blown malignant phenotype, while p*TERT* mutations emerge in intermediate lesions and melanoma in situ. A functional link between *BRAF* and p*TERT* mutations has been demonstrated since the RAS-ERK signaling, in *BRAF*^V600E^ positive melanomas, is critical for regulating active chromatin state and recruitment of RNA polymerase II at mutant p*TERT*. Notably, the mutant p*TERT* is a key substrate downstream of the RAS-ERK pathway [14].

As expected, a combined treatment with dabrafenib and trametinib induced a complete regression of MM skin lesions, which is still maintained (after 12 months of treatment). Whether these inhibitors were also effective against CLL, could not be assessed in our patient, since he had already undergone chemotherapy. However, the use of BRAF/MEK inhibitors, in CLL, might be exploited as new therapeutic approach since *BRAF* exon 15 mutations have been found in ~3% of cases [15].

Seeking for inherited cancer predisposition, we uncovered that the proband’s brother had suffered of multiple MM and both the mother and the sister of breast cancer. However, although genetic counseling suggested a family predisposition (Fig. 2a), we ruled out known germinal mutations of genes involved in these types of hereditary tumors. Interestingly, the patient had a constitutional t(12;17)(p13;p13), not detected in his brother and sister. Even though rare familial CLL were linked to chromosome translocations, the role of this new translocation in collision CLL/MM onset of our cases, could not be determined [16].

## Conclusions

In conclusion, we reported for the first time, a case of collision CLL/MM tumors in a skin lesion [17]. While our molecular-cytogenetic studies proved that specific and distinct genetic events selectively underlie the two lesions, i.e. 11q and 13q deletions for the CLL, and *BRAF*^V600E^ and p*TERT* mutations for the MM, other reports have shown that the two tumors share the same lesion. Although, genetic counseling suggested a familial risk to MM, investigating known gene variations predisposing to MM was not informative. Thus, an inherited condition along with a long lasting story of immune dysregulation might have cooperated in the CLL/MM collision tumour onset of our case.

## Abbreviations

BM: Bone marrow
CLL: Chronic lymphocytic leukemia
DHPCL: Denaturing high performance liquid chromatography
FISH: Fluorescent in situ hybridization
MM: Malignant melanoma
PB: Peripheral blood
PET: Positron emission tomography
PGM: Personal genome machine
PHA: Phyto hemagglutinin
p*TERT*: *TERT* promoter
SUV: Standardized uptake values
UCSC: University of California Santa Cruz
